# Comparative *de novo* Transcriptome Analysis of Two Cultivars With Contrasting Content of Oil and Fatty Acids During Kernel Development in *Torreya grandis*

**DOI:** 10.3389/fpls.2022.909759

**Published:** 2022-06-20

**Authors:** Chi Zhang, Haokai Liu, Hui Zhang, Wanyu Dang, Caihong Zhou, Min Zhang

**Affiliations:** ^1^State Key Laboratory of Subtropical Silviculture, Zhejiang A & F University, Hangzhou, China; ^2^Collaborative Innovation Center for Efficient and Green Production of Agriculture in Mountainous Areas of Zhejiang Province, College of Horticulture Science, Zhejiang A & F University, Hangzhou, China; ^3^Key Laboratory of Quality and Safety Control for Subtropical Fruit and Vegetable, Ministry of Agriculture and Rural Affairs, College of Horticulture Science, Zhejiang A & F University, Hangzhou, China; ^4^Jingning Natural Resources and Planning Bureau, Lishui, China; ^5^Jingning Ecological Forestry Development Center, Lishui, China

**Keywords:** *Torreya grandis*, comparative *de novo* transcriptome, fatty acid (composition), correlation analysis, oil content, kernel development, cultivars

## Abstract

Vegetable oil is an indispensable nutritional resource for human health and mainly characterized by the composition and content of fatty acids (FAs). As a commercial species of gymnosperm, *Torreya grandis* produces oil-rich nuts with high unsaturated fatty acids content in the mature kernels. In this study, two cultivars, *T. grandis* ‘Xifei’ and *T. grandis* ‘Dielsii,’ with distinct oil content were employed to compare the profiles of FAs accumulation during kernel development. The accumulation rate of oil content was significantly different between ‘Xifei’ and ‘Dielsii.’ Besides, the final oil content of ‘Xifei’ (52.87%) was significantly higher than that of ‘Dielsii’ (41.62%) at maturity. The significant differences in main FAs were observed at almost each kernel development stages between the two cultivars. C_16:0_, C_18:1_, and C_20:3_ FA exhibited different accumulation patterns between cultivars. The content and the initiation of accumulation of C_20:3_ FA were different between the two cultivars. To explore the molecular mechanism associated with different content of oil and FAs between two cultivars, *de novo* transcriptome of kernels was compared between ‘Xifei’ (high oil) and ‘Dielsii’ (low oil) at three stages of oil accumulation, respectively. Totally 142,213 unigenes were assembled and 16,379 unigenes with a length of over 1,000 nt were successfully annotated, including 139 unigenes related to FA biosynthesis, elongation, and metabolism. Compared with ‘Dielsii,’ totally 1,476, 2,140, and 1,145 differentially expressed genes (DEGs) were upregulated in ‘Xifei’ at the stage of the initiative, the rapid rise, and the stationary oil accumulation, respectively; the number of downregulated DEGs reached 913, 1,245, and 904, respectively. Relative expressions of 11 DEGs involved in FAs biosynthesis and metabolism were confirmed by RT-qPCR. Abundant differentially expressed transcription factors and pathway DEGs were correlated to oil and FAs according to Pearson’s correlation analysis between transcriptome and metabolites (oil and FAs), suggesting their contributions to the differential oil and FAs between the two cultivars during kernel development of *T. grandis*. To conclude, our findings can provide novel insights into the developmental differences in metabolites and *de novo* transcriptome correlated to lipid accumulation and FA synthesis of kernels between cultivars with contrasting oil deposits and demystify the regulatory mechanism of high oil accumulation in *T. grandis*.

## Introduction

Vegetable oil is indispensable and consumed in the human diet that requires high-quality edible lipids mainly characterized by the composition and proportion of saturated fatty acids (SFAs) and unsaturated fatty acids (UFAs). In most cases, fatty acids (FAs) have evolved during the *de novo* synthesis, elongation, and desaturation process. Most nature oil from plants frequently contains polyunsaturated fatty acids (PUFAs), which contain a long chain of at least 18 carbon atoms and 1 methylene (Lisa et al., 2007) or polymethylene interrupted double bonds (UPIFAs). Sciadonic acid (SA) is a *cis*5, 11, 14-eicosatrienoic acid (Δ5-UPIFAs) with the first unsaturation site at the fifth carbon atom that differs from the structure of other PUFAs (Endo et al., 2009; Xie et al., 2016). Due to the beneficial function for health such as regulating the blood pressure (Sugano et al., 1994; Asset et al., 1999; Endo et al., 2006), anti-inflammatory (Chuang et al., 2009; Chen et al., 2015), and prophylactic impacts on angiosclerosis and obesity (Meng et al., 2020), the plant oils with a certain amount of Δ5-UPIFAs such as SA are essential for dietary modification of human to stem some chronic illnesses.

*Torreya grandis* is a gymnosperm species of six members in *Torreya* genus belonging to the Taxaceae family with wide distribution in South China (Shi et al., 2018). *T. grandis* produces nut-seeds with approximately 50% of dry weight (DW) high-quality oils. Due to dioecious flowers, *T. grandis* possesses abundant variation of seedlings, from which a considerable number of landraces and cultivars were developed and identified with distinct SSR fingerprints (Chen et al., 2020a,b; Meng et al., 2020; Zhang et al., 2021). The commercial value of the rare conifer tree derives mainly from the oil-rich nuts with high UFA content in the mature kernels (Ni et al., 2015; Chen and Jin, 2019a). Great variation in seeds phenotype and qualities of mature nuts exist extensively between cultivars in *T. grandis* (Ni and Shi, 2014; Chen and Jin, 2019b). The oil content of *T. grandis* kernels was reported to range from 51.52 to 55.64% depending on the certain cultivar applied in the test (Ni and Shi, 2014). The content of FAs in mature kernels was significantly different between landraces or cultivars in *T. grandis* (Shi et al., 2018). Oleic (C_18:1_) and linoleic acids (C_18:2_) were dominant FAs in mature kernels based on 10 landraces of *T. grandis*, ranging from 17.62 to 35.11% and 39.77 to 46.06% of total FAs, respectively. The beneficial SA ranged from 9.18 to 18.15% of the total FAs in tested *T. grandis* oil. The difference in the oil content and FA composition influences the flavor of nuts in *T. grandis*.

Underlying the final difference of oil deposition is a complex transcriptional network. Genes that encode enzymes related to *de novo* lipid synthesis pathways, including acetyl-CoA carboxylase, ketoacyl-ACP synthases (KAS), stearoyl-ACP desaturase, acyl-ACP thioesterases (FATA and FATB), and acyl-CoA synthesis (LACS) are characterized and cloned in oil crops such as *Arabidopsis*, *Brassica napus*, and *Zea mays*. In addition, acyltransferases like glycerol-3-phosphate acyltransferase (GPAT), lysophosphatidic acid acyltransferase (LPAT), diacylglycerol acyltransferase (DGAT), or phospholipid: diacylglycerol acyltransferase are responsible for assembly of triacylglycerol (TAG). The oil content could be raised in LPAT overexpression transgenic *Arabidopsis* and *B. napus* (Rao and Hildebrand, 2009). Research showed that DGAT was dominantly responsible for TAG accumulation (Shiu-Cheung and Weselake, 2006; Meng et al., 2009), oil, and oleic acid content (Zheng et al., 2008). Although these pathway genes contributed directly to the distinct biosynthesis of lipids between species and cultivars, little was known about the profiles of regulation of their expression.

The further identification of the transcription factors (TFs) such as *LEAFY COTYLEDON1* (*LEC1*), *WRINKLED1* (*WRI*1), *FUSCA3* (*FUS3*), APETALA2 (AP2)/ETHYLENE RESPONSE FACTOR family, and *ABSCISIC ACID3* (*ABI3*) promote a deeper understanding of oil synthesis during seed development. These TFs were well known in seed storage reserve of nutrients such as lipids, protein, and starch, exhibiting distinct timing patterns, and differentiation of responsibilities that trigger the oil pathway genes (Ruuska et al., 2002). *WRI1* was reported to activate oil accumulation by facilitating the carbon flux by glycolysis (Focks and Benning, 1998; Cernac and Benning, 2004). A homeobox TF GLABRA2 was discovered to prevent the accumulation of seed oil in *Arabidopsis* (Shen et al., 2006). *ABI3*, *AtbZIP10*, and *AtbZIP25* were found for the activation of transcription of storage protein (Lara et al., 2003). In an attempt at developmental control of storage product accumulation, *LEC1* and *LEC2* were found expressed early in transgenic Arabidopsis and subsequently induced *FUS3*, *ABI3*, and *ABI5* expression (Wang et al., 2007), indicating an upstream role of regulation on *FUS3* in oil deposition aided by related increase of FUS3 transcripts and transcripts of enzyme genes involved in oil syntheses during seed development.

Recently, a few studies reported differences in FA composition between landraces and amounts of differentially expressed genes (DEGs) between fruits and vegetable samples (leaf, root, and stem; Wu et al., 2018; Ding et al., 2020), indicating a final profile that coordinates oil deposition at mature kernels in *T. grandis*. Although profiles of oil and FAs are clear at mature kernels, little information is available on dynamic patterns and molecular mechanisms leading to a final difference of oil and FAs with kernel development between cultivars in *T. grandis*.

*Torreya grandis* ‘Xifei’ is a representative cultivar with high oil content and is applied extensively in main producing areas for long periods (He et al., 2016), while *T. grandis* ‘Dielsii’ is well known for rootstock in the propagation of *T. grandis*. A significant difference in oil accumulation was observed in mature kernels between the two cultivars in practice. Due to the large and high heterozygous genome of gymnosperm, a complete genome sequence of *T. grandis* has not been reported up to now. In this study, integrative strategies of lipid measurement and *de novo* transcriptome were employed to compare and understand the dynamic variation of oil and FA biosynthesis and underlying molecular mechanism during kernel development between *T. grandis* ‘Xifei’ and *T. grandis* ‘Dielsii.’

## Materials and Methods

### Samples Collection

Nut seeds with different developmental stages were collected from three individual adult trees of ‘Xifei’ and ‘Dielsii,’ respectively. Sampling was performed on the 408, 426, 438, 448, 458, 468, 475, 485, 498, 510, and 521 day after pollination (DAP). Nut seeds were harvested and immediately preserved at –80°C before RNA preparation, and the corresponding samples for lipid extraction were stored at –20°C in darkness until needed.

### Lipid Extraction and Determine

Frozen-dried powders were individually incubated in petroleum ether at 50°C for 8 h to extract the lipids of kernels harvested with development of the two cultivars. The oil content was shown as a percent of sample DW. The fatty acid composition of the lipid sample was measured by gas chromatography (Waters H-class UPLC, Agilent Technologies, United States) as described in Wolff et al. (1999a) based on a 37-kinds of fatty acid methyl ester standard mixture. The relative content of FA composition was individually determined by the retention time of the FA-methyl ester standard.

### Transcriptome Assembly and Functional Annotation

High-quality RNAs derived from different developmental kernels was prepared and then sequenced on the Illumina HiSeq2500 platform to generate raw reads with reads length of PE125. Following the removal of reads with the adaptor, reads with more than 10% poly-N, and low-quality reads [>50% with Phred quality score (Q) _5 bases] from the raw sequencing data according to in-house Perl scripts (Biomarker Biological Information Technology Co., Ltd., Beijing, China), high-quality clean data were acquired and assembled by Trinity software, in which min_kmer_cov was set 2 and the other parameters are set to default (Grabherr et al., 2011). Briefly, clean reads were assembled into contigs by the de Bruijn graph using k-mer 25 after trials in varying k-mer sizes. Then, mapping of the reads to contigs containing paired-end reads was made to examine contigs of the same transcript and calculate the distance of these contigs. Eventually, the contigs were connected to obtain assembled transcripts unable to be extended on both end. The longest transcripts were considered the unigenes of gene functional annotation.

Gene function annotation was made on the basis of public protein databases: Nr (NCBI non-redundant protein sequences); Nt (NCBI non-redundant nucleotide sequences); KOG (clusters of orthologous groups of proteins); and Swiss-Prot (a protein sequence database with manual annotation and review) with NCBI blast 2.2.28 + with *e*-value 1.0e-5; Pfam (Protein family) *via* HMMER 3.0 package using *e*-value 0.01as well as KO (KEGG Ortholog database) with KEGG Automatic Annotation Server using *e*-value 1.0e-10. According to the annotation for Nr and Pfam databases, the assignment of unigenes was made in Gene Ontology (GO) with Blast2GO 2.5 (Conesa and Gotz, 2008) using *e*-value 1.0e-6. GO classification was distributed with the Web Gene Ontology Annotation Plot, which included biological process, molecular function, and cellular component.

The calculation method of the fragments per kilobase per million fragments (FPKM) was employed to determine the transcript abundance of each gene (Mortazavi et al., 2008). The DEGs between compared groups were evaluated with the threshold of FC (fold change) >2 and FDR (false discovery rate) <0.01 (Benjamini et al., 2001).

### Reverse Transcript Quantitative PCR

Total RNA was individually extracted from kernels of two cultivars sampled at 408, 438, 458, 475, 510, and 521 DAP with a modified method of RNAiso Plus (Takara Bio Inc, Japan), respectively. The cDNA template of each sample for reverse transcription quantitative PCR (qRT-PCR) was prepared by EasyScript One-Step gDNA removal and cDNA synthesis supermix (TRANSGEN). Pathway genes and TFs involved in FA metabolism were chosen for validation using qRT-PCR. Specific primers of qRT-PCR were obtained with Primer Quest^1^ based on the assembly sequences in the transcriptome. The *Actin* gene was used as reference gene, and the primer sequences were described in the Supplementary Table 9. SYBR Premix Ex Taq™II (Takara) was employed in qRT-PCR with 20 μl volumes on the Roche Light Cycler480 system (Roche Diagnostics, CA, United States). All samples were tested in triplicate. Each reaction was conducted as below: 95°C for 30 s, followed by 40 cycles at 95°C for 5 s, and 60°C for 34 s.

### Statistical Analysis

The determinations of metabolites were performed thrice and error bars in presented figures stand for SD. Statistical analysis was performed with SPSS 20.0. Data comparison was made with one-way analysis of variance (ANOVA), and differences among tested metabolites of two cultivars were evaluated with Duncan’s multiple-range tests at *p* < 0.05. Pearson correlation coefficient (*r*) was calculated for correlation analysis on DEGs and metabolites.

## Results

### Dynamic Changes in the Oil Content and Fatty Acids Composition Between *T. grandis* ‘Xifei’ and *T. grandis* ‘Dielsii’ During Kernel Development

**FIGURE 1:**
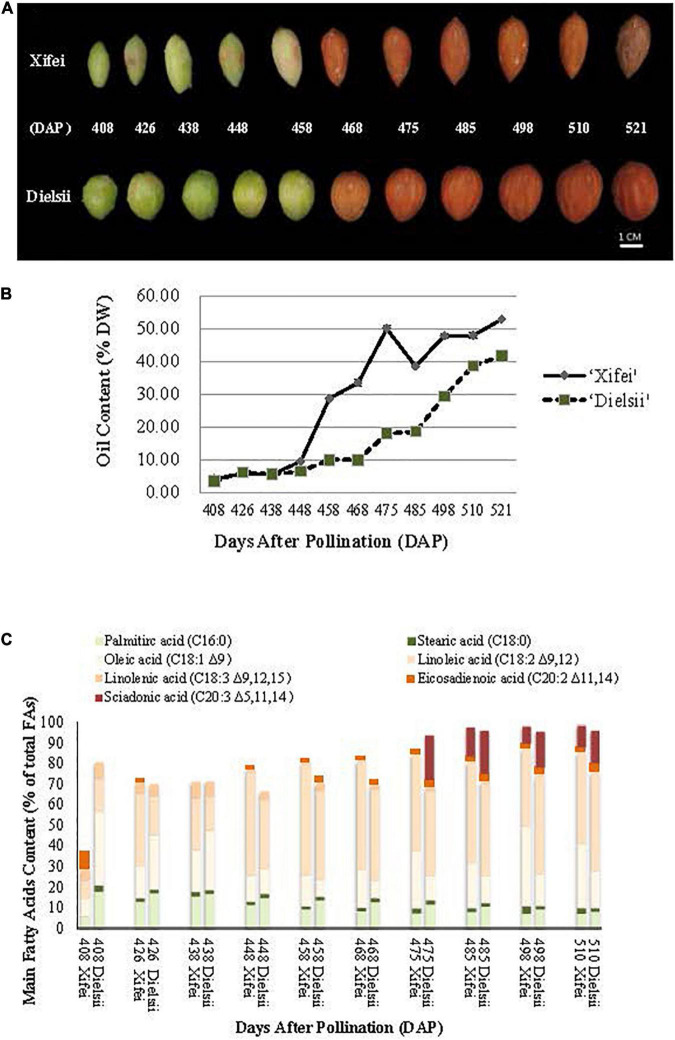
Developing nut-seeds, the content of oil, and FAs in *Torreya grandis* ‘Xifei’ and *T. grandis* ‘Dielsii,’ respectively. DAP, day after pollination. **(A)** Developing nut-seeds of two cultivars at each sampling time. **(B)** Oil content of ‘Xifei’ (black dots) and ‘Dielsii’ (white dots) at each sampling time, respectively. Values of oil content were expressed as the percentage of dry weight (%DW) of kernels. **(C)** The content of FA composition in ‘Xifei’ and ‘Dielsii’ at each sampling time was presented as a percentage of total FAs (%), respectively.

Concerning 17 months for ripening duration in kernels, nut-fruits derived from *T. grandis* ‘Xifei’ and *T. grandis* ‘Dielsii’, respectively, were sampled from 408 to 521 DAP (Figure 1A) to characterize the temporal variation of the oil content and FA composition during kernel development. Generally, the oil content was raised gradually with kernel development (Figure 1B and Supplementary Table 1). During the initiation phase, no difference in the oil content was observed and the curve line of the oil content almost overlapped from 408 to 438 DAP between the two cultivars. A wider gap occurred in the subsequent 10 days, and a significant difference in the oil content was observed at 448 DAP. Then, the oil content in ‘Xifei’ raised more rapidly and exceeded that in ‘Dielsii.’

When the fruits get mature at 521 DAP, the oil content is distinct between ‘Xifei’ and ‘Dielsii,’ reaching 52.87 ± 0.19% and 41.62 ± 0.23%, respectively. To summarize, the oil content of ‘Xifei’ was raised more rapidly and higher than that of ‘Dielsii’ during kernel development, suggesting a differentially regulatory mechanism underlying the phenotype of oil between two cultivars of *T. grandis*.

Meanwhile, FA composition and content of ‘Xifei’ and ‘Dielsii’ were measured and compared during the whole development of kernels (Figure 1C and Supplementary Table 2). Generally, palmitic acid (C_16:0_) was the dominant SFA in both ‘Xifei’ and ‘Dielsii.’ C_16:0_ FA of ‘Xifei’ fell down after a transitory rise before 438 DAP, while C_16:0_ FA of ‘Dielsii’ kept plummeting during the whole development of kernels. C_16:0_ FA of ‘Xifei’ was significantly less than that of ‘Dielsii’ at each stage of kernel development. C_18:0_ FA of two cultivars was less than 4% during the whole development. UFAs recognized and studied by the current research included oleic acid (C_18:1_), linoleic acid (C_18:2Δ9,12_ and C_18:3Δ9,12,15_), eicosadienoic acid (C_20:2Δ11,14_), and SA (C_20:3Δ5,11,14_). In general, dominant UFA was C_18:2Δ9,12_, C_18:1_, and C_20:3Δ5,11,14_ in both cultivars, and total main UFAs were raised with kernel growing. Besides, the content of UFAs in ‘Xifei’ surpassed that in ‘Dielsii’ at almost all stages of kernel development and reached 88.27% significantly higher than that in ‘Dielsii’ (85.88%) at maturation. Among three main UFAs, C_18:2_ accounted for the majority of UFA in both cultivars, and the significant differences in C_18:2_ content were observed between cultivars at each developmental phase, which ranged from 16.69 ± 2.65% to 47.69 ± 0.7% and 9.10 ± 0.02% to 43.91 ± 0.15% in ‘Dielsii’ and ‘Xifei’ during kernel development, respectively. C_18:1_ is the secondary UFA. The content of C_18:1_ in ‘Dielsii’ was significantly exceeded that in ‘Xifei’ during the initiation phase. However, C_18:1_ FA of ‘Xifei’ surpassed that of ‘Dielsii’ in the subsequent development of kernels. UFAs with 20 carbon atoms mainly include C_20:2Δ11,14_ and C_20:3Δ5,11,14_ in this study. C_20:2Δ11,14_ FA contributed approximately 2% in ‘Xifei’ and 2–4% in ‘Dielsii’ to total FAs during the maturation of kernels. C_20:3Δ5,11,14_ FA accounted for 8–14.16% and 2.17–21.76% of total FAs in ‘Xifei’ and ‘Dielsii.’ C_20:2 Δ11,14_ had been accumulated almost from the initiation phase, whereas C_20:3Δ5,11,14_ was not detected until 475 and 485 DAP in ‘Dielsii’ and ‘Xifei,’ respectively. When the kernels developed into maturation, the content of C_20:3Δ5,11,14_ FA reached 15.76 ± 0.28% in ‘Dielsii’ and 10.56 ± 0.08% in ‘Xifei.’ In conclusion, a significant difference was obtained in C_16:0_, C_18:1_, and C_20:3Δ5,11,14_ FA between two cultivars during kernel development.

### *De novo* Assembly Sequencing and Annotations

To decipher the molecular mechanism responsible for the difference of oil and FAs between ‘Xifei’ and ‘Dielsii’ during kernel development, comparative transcriptome analysis was further performed between two cultivars at 438 DAP (initiation), 475 DAP (rapid rise), and 510 DAP (stationary) in accordance with distinct oil content in lipid measurement, respectively. Over 6.0 Gb of clean data were obtained and Q30 is more than 90% in each sample (Supplementary Table 3). The *de novo* transcriptome assembly was conducted to obtain 142,213 non-redundant unigenes and 22,976 unigenes with over 1 kb in length (Supplementary Figure 1). Annotation of unigenes was carried out based on the best-matched known sequences. The best-matched species of the unigenes in *T. grandis* successively included *Picea sitchensis* (22%), *Amborella trichopoda* (7%), *Nelumbo nucifera* (6%), *Vitis vinifera* (5%), and *Prunus persica* (3%; Supplementary Figure 2A).

Functional classification was carried out to better understand the unigenes on biological function and interaction in development. A total of 6,485 unigenes were allocated to 119 pathways, and 139 unigenes were related to fatty acid biosynthesis, elongation, and metabolism pathways. A total of 17,783 unigenes were annotated into 52 subgroups in accordance with GO function terms including biological process, cellular component, and molecular function subgroups (Supplementary Table 4 and Supplementary Figure 2B). The dominating biological processes were metabolic, cellular, and single-organism processes. The top molecular function was catalytic activity and binding.

### Differentially Expressed Genes Involved in Fatty Acid Biosynthesis and Metabolism During Kernel Development Between Two Cultivars

**FIGURE 2:**
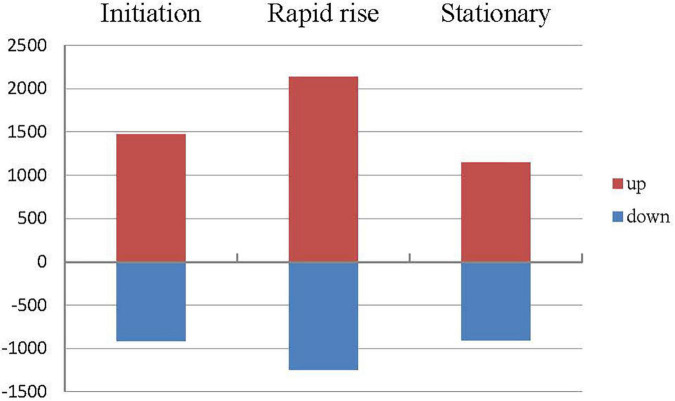
Differentially expressed genes (DEGs) related to fatty acids metabolism between ‘Xifei’ and ‘Dielsii’ at initiation, rapid rise and stationary stage of kernel development, respectively.

A total of 5,683 DEGs were obtained in comparison between cultivars (Supplementary Table 5). Compared to ‘Dielsii,’ a total of 1,476, 2,140, and 1,145 DEGs were upregulated during the stage of initiation, rapid rise, and the stationary oil accumulation in ‘Xifei,’ respectively. DEGs with significantly decreased expression sum up to 913, 1,246, and 904 at three stages in ‘Xifei,’ respectively, (Figure 2). Interestingly, a total of 177 differentially expressed transcription factors (DETFs) were captured between cultivars, which contained 13 DETFs involved in three stages, 41 DETFs activated or suppressed at two stages, and 123 DETFs with functions only at one stage of kernel development.

By comparing development stages, DEGs associated with development were 5,933 and 5,406 in ‘Xifei’ (Supplementary Table 6) and ‘Dielsii’ (Supplementary Table 7), respectively. In ‘Xifei,’ DEGs of upregulation and downregulation were up to 1,236 and 1,769 when the oil content increased rapidly. A total of 714 and 873 DEGs were increased and decreased in expression when oil was steadily stored, respectively. Similarly, in ‘Dielsii,’ the up- and down-regulated DEGs reached 1,637 and 2,065 when oil accumulated rapidly, while 1,316 and 1,012 DEGs were up and down expressed during the stationary phase of oil accumulation. In addition, more DETFs were found in the comparison between stages of kernel development, which were captured by 213 DETFs in ‘Xifei’ and 206 DETFs in ‘Dielsii.’

**FIGURE 3:**
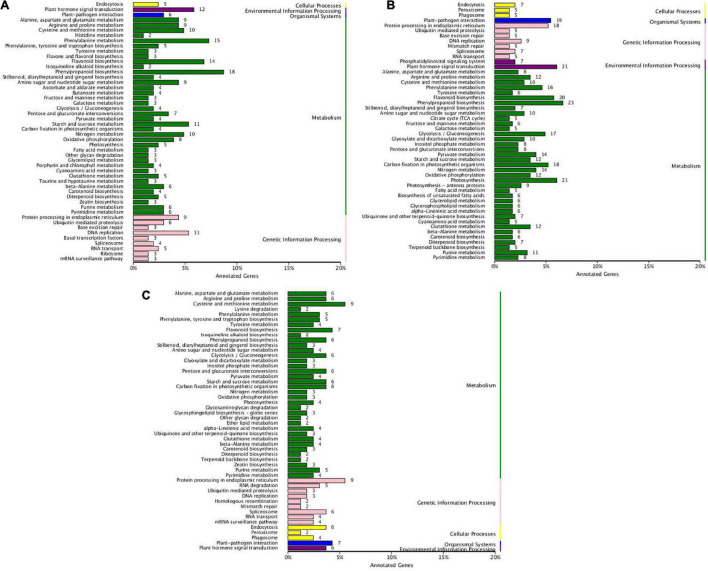
KEGG enrichment of DEGs between *T. grandis* ‘Xifei’ and *T. grandis* ‘Dielsii’ at the development of the initiative **(A)**, rapid rise **(B)**, and stationary **(C)** oil accumulation.

KEGG pathway of DEGs was analyzed and the comparison between cultivars was focused on (Figure 3). Abundant DEGs between cultivars were dominantly involved in metabolism pathways (Supplementary Table 8). DEGs involved in fatty acid metabolism were enriched at both initiation (Figure 3A) and rapid rise stage (Figure 3B). DEGs involved in the biosynthesis of UFAs were enriched only at the rapid rise stage. More DEGs were found in the rapid rise phase of oil production between cultivars (Table 1). Besides, up- or down-streamed pathways of fatty acid biosynthesis, such as glycolysis, pyruvate metabolism, glycerolipid metabolism, and glycerophospholipid metabolism, were specifically enriched in the rapid rise stage when compared between cultivars.

**TABLE 1:** DEGs involved in pathways related to fatty acid biosynthesis and metabolism between *T. grandis* ‘Xifei’ and *T. grandis* ‘Dielsii’.

KEGG pathway	Swissprot_ annotation	Initial stage			Rapid rise			Stationary		
		FDR	log2FC	Regulated	FDR	log2FC	Regulated	FDR	log2FC	Regulated
**Biosynthesis of unsaturated fatty acids (6)**										
c99245.graph_c0	Protein ODORANT1 (ODO1)	0.002124202	–1.71072342	Down	–	–	–	–	–	–
c90340.graph_c0	Transcription factor RAX2	3.76E-08	–1.1885191	Down	–	–	–	–	–	–
c92934.graph_c0	Transcription factor MYB86	4.36E-08	–2.40718869	Down	–	–	–	–	–	–
c94730.graph_c1	Nuclear transcription factor Y subunit C-1	0.006910842	–1.21805909	Down	–	–	–	–	–	–
c85671.graph_c0	–	0.000918138	1.44437094	Up	0.003367907	1.853099391	Up	1.86E-05	2.28933565	Up
c98417.graph_c0	NADPH–cytochrome P450 reductase	–	–	–	0.002346744	5.974724421	Up	–	–	–
**Fatty acid biosynthesis (2)**										
c98678.graph_c0	–	–	–	–	–	–	–	3.49E-05	–1.20285232	Down
c99245.graph_c0	GRAS family transcription factor	–	–	–	0.000103453	3.378469799	Up	–	–	–
**Fatty acid metabolism (8)**										
c43899.graph_c0	–	1.98E-06	–2.89845052	Down	–	–	–	–	–	–
c101043.graph_c0	26S protease regulatory subunit 6B homolog	–	–	–	2.44E-13	2.426790509	Up	–	–	–
c91980.graph_c0	–	–	–	–	–	–	–	0.005208433	–1.21416289	Down
c94509.graph_c0	–	–	–	–	2.48E-05	1.337339018	Up	–	–	–
c89303.graph_c0	F-box/kelch-repeat protein At5g43190	–	–	–	9.65E-20	–1.97495432	Down	–	–	–
c90340.graph_c0	MAR-binding filament-like protein 1 (MFP1)	–	–	–	1.52E-11	–1.31606418	Down	–	–	–
c99307.graph_c0	Probable WRKY transcription factor 11 (WRKY11)	0.00384533	1.57568211	Up	–	–	–	–	–	–
c81605.graph_c0	(R,S)-reticuline 7-O-methyltransferase (PSOMT1)	0.001091065	2.283869688	Up	2.61E-13	4.464544002	Up	–	–	–
**Glycerolipid metabolism (7)**										
c69013.graph_c0	Diacylglycerol O-acyltransferase 1-like	–	–	–	0.000733551	–1.89319743	Down	–	–	–
c101495.graph_c0	UDP-sulfoquinovose synthase, chloroplastic	5.55E-07	–2.71208322	Down	–	–	–	–	–	–
c72149.graph_c0	Alpha-galactosidase	–	–	–	1.15E-12	1.452865054	Up	6.99E-05	1.075970917	Up
c80383.graph_c0	–	–	–	–	0.001237479	1.285968585	Up	–	–	–
c89559.graph_c0	Hypothetical protein AMTR	–	–	–	1.95E-06	–1.08251029	Down	–	–	–
c81605.graph_c0	Aldehyde dehydrogenase family 3 member F1	0.001091065	2.283869688	Up	2.61E-13	4.464544002	Up	–	–	–
c92777.graph_c0	–	3.24E-05	1.342914101	Up	4.48E-15	1.610510221	Up	–	–	–
**Glycerophospholipid metabolism (7)**										
c102929.graph_c0	Hypothetical protein AMTR	–	–	–	1.03E-08	Inf	Up	–	–	–
c89559.graph_c0	Hypothetical protein AMTR	–	–	–	1.95E-06	–1.08251029	Down	–	–	–
c86728.graph_c0	Non-specific phospholipase C2	–	–	–	–	–	–	6.54E-06	1.724029337	Up
c92777.graph_c0	–	3.24E-05	1.342914101	Up	4.48E-15	1.610510221	Up	–	–	–
c101355.graph_c0	–	–	–	–	2.95E-14	1.46223635	Up	5.36E-08	1.695178981	Up
c91802.graph_c0	Non-specific phospholipase C6	–	–	–	2.30E-11	–1.4210226	Down	–	–	–
c91903.graph_c0	Phospholipase D	–	–	–	0.00147208	1.307370985	Up	–	–	–

**FIGURE 4:**
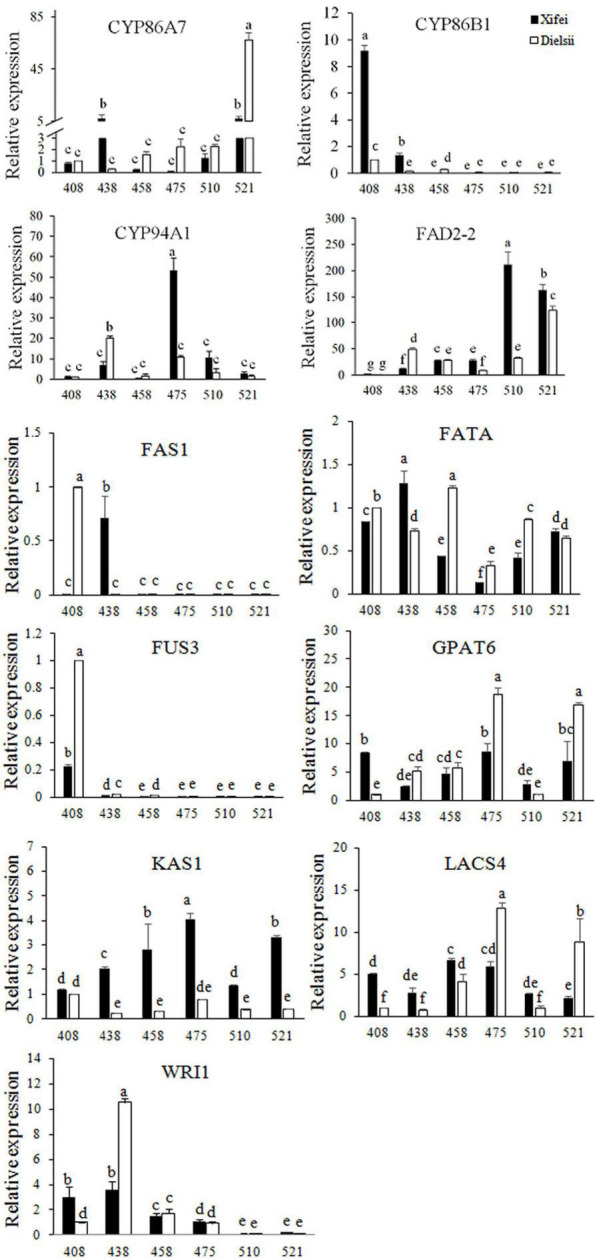
RT-qPCR of 11 DEGs related to fatty acid biosynthesis and metabolism measured during development. Relative expression of DEGs was shown with means of triplicates. Samples were collected at 408, 438, 458, 475, 510, and 521 days after pollination (DAP). The dark square represents *T. grandis* ‘Xifei.’ White square represents *T. grandis* ‘Dielsii.’ The significant difference of the DEG expression between development stages were labelled with a to g according to Duncan’s multiple comparison test (*P* < 0.05).

Differentially expressed genes expression related to fatty acid biosynthesis and the metabolism was verified in kernel development (Figure 4 and Supplementary Table 9), which included 3 *CYP* family genes, *FAD2-2*, *FAS1*, *KAS1*, *GPAT6*, *LACS4*, *FUS3*, *WRI1*, and *FATA*. The expression patterns of DEGs were almost consistent with the transcriptome.

### Integrative Analyses of Fatty Acid and Transcriptome

**FIGURE 5:**
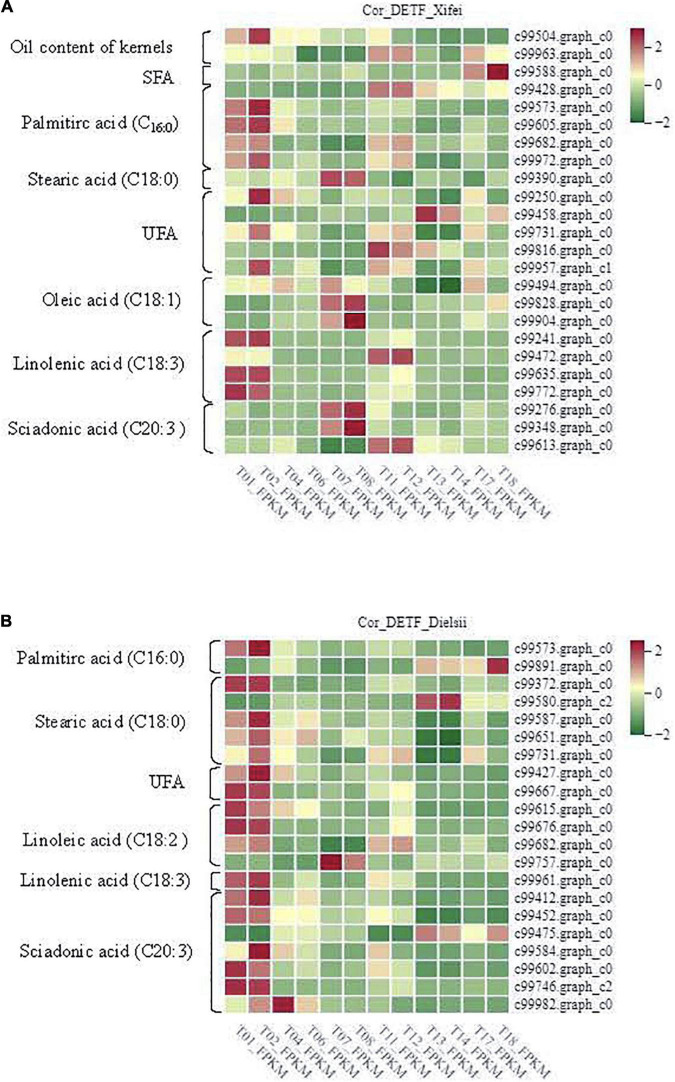
Differentially expressed transcription factors (DETFs) were correlated to metabolites at different stages of kernel developments. **(A)** Samples termed as T01, T02, T04, T06, T07, and T08 represent the stage of the initiative, rapid and stationary oil accumulation in kernels of *T. grandis* ‘Xifei,’ respectively. **(B)** Samples termed as T011, T12, T13, T14, T17, and T18 represent the stage of the initiative, rapid and stationary oil accumulation in kernels of *T. grandis* ‘Dielsii,’ respectively. The correlated metabolite (*p* < 0.01) was listed in the left column.

To further identify the related DEGs contributing to the oil and FAs differences, the analyses of the Pearson Correlation Coefficient (*r*) were carried out with the total available 5,683, 5,933, and 5,406 non-redundant DEGs identified from the comparison of cultivars and development in ‘Xifei’ and ‘Dielsii,’ respectively, (Supplementary Tables 5–7). A total of 922 and 574 DEGs (*p* < 0.01) derived from the comparison of the two cultivars (cultivar-DEGs) were closely associated with the synthesis and metabolism of oil and FAs in ‘Xifei’ and ‘Dielsii,’ respectively, (Supplementary Tables 10,11). The numbers of cultivar-DEGs correlated to different types of FA were extremely different between cultivars. Totally 24 and 21 correlated DETFs were identified in ‘Xifei’ and ‘Dielsii,’ respectively, (Table 2), and the expression profile of DETFs was individually correlated to different FAs according to the FPKM value of transcriptome (Figure 5 and Supplementary Table 12). Concerning distinct oil content between cultivars, WRKY45 (c99504.graph.c0, *r* = –0.99997, and *p* = 0.00482) and ERF017 (c99963.graph_c0, *r* = –0.99996, and *p* = 0.00599) were involved in oil content of kernels with negative coefficient in ‘Xifei,’ exhibiting downregulated expression with kernel development in ‘Xifei.’ A total of 15 DETFs were correlated to syntheses of unsaturated FAs in ‘Xifei,’ containing 5 involved in UFA, 3 DETFs in C_18:1_ FA, 4 DETFs in C_18:3_ FA, and 3 in C_20:3_ FA. Among three DETFs of MYB type termed as GTL1, two exhibited positive correlation to C_18:1_ FA (c99828.graph_c0, *r* = 1.00000, and *p* = 0.00154) and C_20:3_ FA (c99348.graph_c0, *r* = 0.99998, and *p* = 0.00413), and one was negatively correlated to UFA (c99816.graph_c0, *r* = –0.99997, and *p* = 0.00525). FUS3 (c99613.graph_c0) was involved in C_20:3_ FA syntheses in ‘Xifei’ with negative coefficient (*r* = –0.99993, *p* = 0.00766). Given that C_20:3_ FA in ‘Dielsii’ was significantly different from that in ‘Xifei,’ a total of 6 DETFs were concerned in C_20:3_ FA syntheses with negative correlation, and only one DETF was positively correlated (ERF RAP2-3, *r* = 0.99988, and *p* = 0.009995) and significantly upregulated with the development of kernels in ‘Dielsii.’

**TABLE 2:** Differentially expressed transcription factors (DETFs) between cultivars and their correlation to metabolites.

Cultivar	Gene ID	Annotation	Metabolites	Cor_r	*p*-value
*T. grandis* ‘Xifei’	c99504.graph_c0	WRKY45	Oil content of kernels(%)	–0.99997	0.00482
	c99963.graph_c0	ERF017	Oil content of kernels(%)	–0.99996	0.00599
	c99588.graph_c0	ERFABR1	SFA	–0.99991	0.00841
	c99428.graph_c0	GATA22	Palmitirc acid (C16:0)	0.99990	0.00905
	c99573.graph_c0	ERF018	Palmitirc acid (C16:0)	0.99999	0.00255
	c99605.graph_c0	GATA2	Palmitirc acid (C16:0)	0.99995	0.00667
	c99682.graph_c0	PLATZ	Palmitirc acid (C16:0)	0.99997	0.00508
	c99972.graph_c0	MADS6	Palmitirc acid (C16:0)	0.99993	0.00760
	c99390.graph_c0	MYB (APL)	Stearic acid (C18:0)	0.99989	0.00937
	c99250.graph_c0	ARF31	UFA	–0.99988	0.00992
	c99458.graph_c0	bHLH66	UFA	0.99999	0.00291
	c99731.graph_c0	PLATZ	UFA	–0.99999	0.00321
	c99816.graph_c0	MYB (GTL1)	UFA	–0.99997	0.00525
	c99957.graph_c1	MYB23	UFA	–0.99998	0.00415
	c99494.graph_c0	ERF1A	Oleic acid (C18:1 Δ9)	0.99999	0.00263
	c99828.graph_c0	MYB (GTL1)	Oleic acid (C18:1 Δ9)	1.00000	0.00154
	c99904.graph_c0	ERF003	Oleic acid (C18:1 Δ9)	0.99991	0.00860
	c99241.graph_c0	WOX9	Linolenic acid (C18:3 Δ9,12,15)	0.99988	0.00993
	c99472.graph_c0	bHLH95	Linolenic acid (C18:3 Δ9,12,15)	0.99998	0.00450
	c99635.graph_c0	ICE1	Linolenic acid (C18:3 Δ9,12,15)	0.99999	0.00317
	c99772.graph_c0	UVR8	Linolenic acid (C18:3 Δ9,12,15)	0.99993	0.00754
	c99276.graph_c0	MYB	Sciadonic acid (C20:3 Δ5,11,14)	0.99991	0.00866
	c99348.graph_c0	MYB (GTL1)	Sciadonic acid (C20:3 Δ5,11,14)	0.99998	0.00413
	c99613.graph_c0	FUS3	Sciadonic acid (C20:3 Δ5,11,14)	–0.99993	0.00766
*T. grandis* ‘Dielsii’	c99573.graph_c0	ERF018	Palmitirc acid (C16:0)	0.99994	0.00688
	c99891.graph_c0	ODORANT1	Palmitirc acid (C16:0)	–0.99989	0.00935
	c99372.graph_c0	bHLH49	Stearic acid (C18:0)	1.00000	0.00185
	c99580.graph_c2	bHLH118	Stearic acid (C18:0)	–1.00000	0.00152
	c99587.graph_c0	bHLH30	Stearic acid (C18:0)	0.99988	0.00997
	c99651.graph_c0	ERF3	Stearic acid (C18:0)	0.99991	0.00865
	c99731.graph_c0	PLATZ	Stearic acid (C18:0)	1.00000	0.00196
	c99427.graph_c0	FAMA	UFA	–0.99998	0.00439
	c99667.graph_c0	ERF RAP2-3	UFA	–0.99995	0.00645
	c99615.graph_c0	DIVARICATA	Linoleic acid (C18:2 Δ9,12)	–0.99988	0.00987
	c99676.graph_c0	ERF3	Linoleic acid (C18:2 Δ9,12)	–0.99989	0.00960
	c99682.graph_c0	PLATZ	Linoleic acid (C18:2 Δ9,12)	–0.99994	0.00669
	c99757.graph_c0	IIB	Linoleic acid (C18:2 Δ9,12)	0.99990	0.00907
	c99961.graph_c0	ILI6	Linolenic acid (C18:3 Δ9,12,15)	0.99999	0.00273
	c99412.graph_c0	bHLH110	Sciadonic acid (C20:3 Δ5,11,14)	–1.00000	0.00001
	c99452.graph_c0	GATA5	Sciadonic acid (C20:3 Δ5,11,14)	–1.00000	0.00013
	c99475.graph_c0	ERF RAP2-3	Sciadonic acid (C20:3 Δ5,11,14)	0.99988	0.009995
	c99584.graph_c0	bHLH74	Sciadonic acid (C20:3 Δ5,11,14)	–0.99990	0.00921
	c99602.graph_c0	bHLH93	Sciadonic acid (C20:3 Δ5,11,14)	–0.99994	0.00679
	c99746.graph_c2	BRX	Sciadonic acid (C20:3 Δ5,11,14)	–0.99995	0.00621
	c99982.graph_c0	HY5	Sciadonic acid (C20:3 Δ5,11,14)	–1.00000	0.00065

**FIGURE 6:**
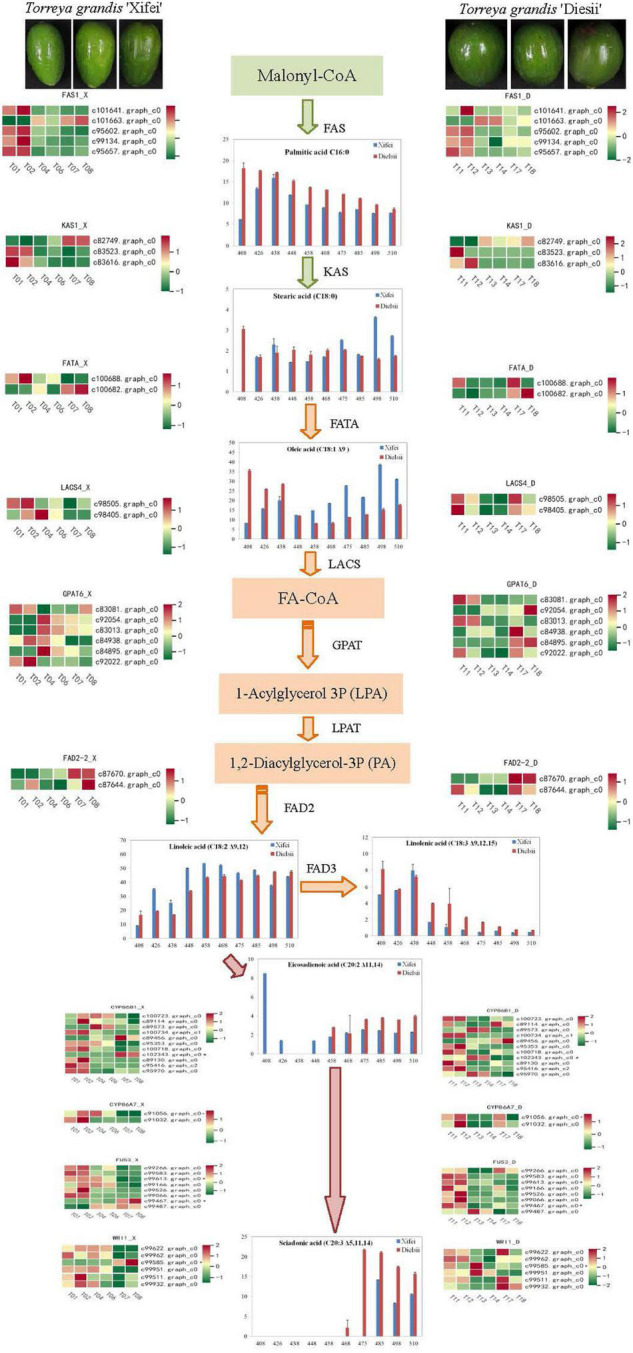
Differential expression of unigenes associated with fatty acid biosynthesis with kernel development between *T. grandis* ‘Xifei’ (left) and *T. grandis* ‘Dielsii’ (right). Enzyme, unigene, and expression profiles are individually indicated at each primary step of biosyntheses. Colorful grids ranging from green to red in the row indicated the FPKM value of each unigene at the different development stages of kernels. Unigenes with significant correlation to C_20:3_ FA were indicated with an asterisk (*) beside the sequence ID.

To better understand the difference in regulation on the pathway of FA syntheses, DEGs were identified based on references and paralleled with the pathway of FA biosynthesis. The pathway of FA syntheses was initiated from malony-CoA and subsequently catalyzed by enzymes of FA syntheses. The profiles of DEGs were shown in the form of a heatmap (Figure 6 and Supplementary Table 13). Given that C_16:0_ FA was differentially produced between two cultivars, the expression of FAS was upregulated at the initial development of kernels development and responsible for the nascent rise of C_16:0_ FA in ‘Xifei,’ whereas the declined expression of FAS accounted for C_16:0_ FA in ‘Dielsii.’ When C_18:0_ FA was converted into C_18:1_ FA, FATA (c100682.graph_c0) was upregulated by kernel development in ‘Xifei’ and ‘Dielsii,’ while FATA (c100688.graph_c0) was upregulated only at nascent syntheses of FA in ‘Xifei,’ indicating a different rule accounting for the more accumulation of C18:1 between two cultivars. Given that a total of 10 DETFs were significantly associated with C_20:3_ FA syntheses, the expression of these DETFs was different between cultivars during kernel development. Eight DETFs were upregulation at the early stage of kernel development in ‘Xifei.’ Only one DETF (c99475.graph_c0) was specifically upregulated at kernel maturation in ‘Dielsii,’ whereas the expression of c99613.graph_c0 was upregulated at the early stage of ‘Dielsii,’ suggesting the differential regulation rules on the deposition of C_20:3_ FA between ‘Xifei’ and ‘Dielsii.’

## Discussion

### Dynamic Characters and Difference of Oil and Fatty Acid Composition During Kernel Development in *T. grandis* ‘Xifei’ and *T. grandis* ‘Dielsii’

*Torreya grandis* was one of the rare conifer trees in China, and was famous for the high content of oil in dry nuts. Although the final profiles of oil and FA have been reported in a few published reports (Niu et al., 2011; Ni and Shi, 2014; Shi et al., 2018), the rules of differential accumulation with kernel development remained unclear. In the current study, oil deposition was compared between two cultivars, ‘Xifei’ and ‘Dielsii,’ suggesting a dynamic profile of oil and FA biosyntheses during kernel development. The oil content of the two cultivars was within the variant scale in previous reports (Shi et al., 2018). The oil content of ‘Xifei’ rose more rapidly with kernel growth and was significantly higher than that of ‘Dielsii’ at mature, suggesting a more efficient mechanism of regulation and biosynthesis of lipids in ‘Xifei.’ Interestingly, FA syntheses were significantly different between the two cultivars. The content of C_18:1_ FA in ‘Xifei’ had been increased more rapidly than ‘Dielsii’ after 448 DAP responsible for the higher final deposition of C_18:1_ FA in ‘Xifei.’ C_20:3_ FA (especially refers to SA) was reported to exist in almost all coniferophyte families (Wolff et al., 2001). Besides, *T. grandis* was a rare species that contain high levels of C_20:3_ FA (Niu et al., 2011). Although different final deposition of C_20:3_ FA has been reported between landraces, the developmental changes of C_20:3_ FA were poorly understood. In this study, accumulation of C_20:3_ FA was initiated at 468 DAP in ‘Dielsii’ and 485 DAP in ‘Xifei,’ indicating a novel rule of biosyntheses different from other FAs and a delayed deposition in ‘Xifei’ accounting for the final lower content of C_20:3_ FA. It was reported that the C_20:3_ FA presence and its content varied between varieties (Ni and Shi, 2014; He et al., 2016; Wang et al., 2016; Shi et al., 2018), which are depending on certain cultivars of *T. grandis* and promising a potential correlation to the function of PUFA. As is well known for rootstock, ‘Dielsii’ has a lower content of oil and UFAs but higher content of C_20:3_ FA, suggesting a smart strategy that enrichment of more beneficial polyUFAs such as C20:3 at the expanse of reduction in the oil content in non-edible cultivars of *T. grandis*.

### Comprehensive Regulation of Differentially Expressed Genes Involved in Fatty Acid Biosynthesis

The supply of FAs is a factor restricting oil accumulation in embryo development (Bao and Ohlrogge, 1999). Based on the comparison of DEGs between two cultivars, the most enriched KEGG pathways were glycolysis and oxidative phosphorylation, implying a difference in supplies of substances of fatty acids biosynthesis or processing between ‘Xifei’ and ‘Dielsii.’

Acyl carrier protein (ACP) is crucial for elongation of acyl chain in *de novo* FA synthesis in plants. Acyl-ACP thioesterases are required to catalyze acyl-ACP hydrolysis to discharge free FA (Dussert et al., 2013). FatA and FatB are two classes of Acyl-ACP thioesterases (Jones et al., 1995). FatA enzymes preferentially hydrolyzed18:1-ACP whereas FatB usually catalyzed saturated acyl-ACP (Dussert et al., 2013). Temperate oil crops, such as Arabidopsis, are typically accepted to accumulate low saturated FAs in accordance with the low level of FatB transcripts (Troncoso-Ponce et al., 2011). *T. grandis* is a precious conifer tree with high lipid-rich nut-fruits, the unsaturated FAs of *T. grandis* usually occupy over 85% approximately (Shi et al., 2018). In this study, two DEGs encoding FATA, c100688.graph_c0 in ‘Xifei’ and c100682.graph_c0 in ‘Dielsii,’ differentially expressed with kernel development. FATA (c100682.graph_c0) was upregulated during kernel development, which was in contrast to the lower accumulation of C_18:1_ FA in ‘Dielsii.’ FATA (c100688.graph_c0) in ‘Xifei’ was upregulated at initiation and then declined with the kernel growing, associating with the nascent rise of C_18:1_ FA content in ‘Xifei.’ Interestingly, the expression of three DETFs correlated to C_18:1_ FA was consistent with the presence of C_18:1_ FA in ‘Xifei’ and ‘Dielsii’ (Figure 6). In this study, two DEGs encoding FATA were expressed differentially during kernel development, revealing a novel regulation mechanism of C_18:1_ FA syntheses in *T. grandis*.

C_20:3_ FA (SA) was first found in *Taxus cuspidate* (Japanese yew; Itabashi and Takagi, 1982), mainly distributed in gymnosperm species with wide content variation between family, genus, or even the species (Aitzetmuller, 1995; Wolff et al., 1999a,b; Berger et al., 2002). Concerning the outstanding benefits to health, C_20:3_ FA was widely studied in extraction, purification, characteristics, and pharmacodynamics (Tanaka et al., 2001; Berger et al., 2002). Subsequently, functional characterization of candidate genes encodingΔ5-desaturase in transgenic Arobidopsis was carried out (Sayanova et al., 2007). Recently, an attempt on the exploration of genes involved in the biosyntheses of C_20:3_ FA was performed in *T. grandis* using mature seeds and vegetable organs (leaf, stem, and root; Wu et al., 2018). However, the regulation mechanism of C_20:3_ FA biosyntheses during kernel development remains unclear. In this study, the DEGs between two cultivars with contrasting content of C_20:3_ FA were explored by *de novo* transcriptome during the whole maturation of kernels. Combined with correlation analyses, abundant DEGs correlated to C_20:3_ FA deposition were captured (Supplementary Tables 10,11), including particularly 10 DETFs with significantly different expressions between the two cultivars and responsible for different content of C_20:3_ FA, promising a regulation mechanism in the process of C_20:3_ FA biosyntheses.

## One Sentence Summary

Lipid synthesis and *de novo* transcriptome were compared between two cultivars during kernel development in *Torreya grandis*.

## Data Availability Statement

The datasets presented in this study can be found in online repositories. The names of the repository/repositories and accession number(s) can be found in the article/Supplementary Material.

## Author Contributions

CZ, HL, and MZ wrote the manuscript. CZ and MZ analyzed the data. HZ, WD, and CHZ performed the experiments. All authors contributed to the article and approved the submitted version.

## Conflict of Interest

The authors declare that the research was conducted in the absence of any commercial or financial relationships that could be construed as a potential conflict of interest.

## Publisher’s Note

All claims expressed in this article are solely those of the authors and do not necessarily represent those of their affiliated organizations, or those of the publisher, the editors and the reviewers. Any product that may be evaluated in this article, or claim that may be made by its manufacturer, is not guaranteed or endorsed by the publisher.

## Footnotes

1
https://www.genscript.com/ssl-bin/app/primer

## Supplementary Material

Supplementary Figure 1 Length distribution of unigenes with over 1 kb in length.

Supplementary Figure 2 The best-matched species of the unigenes **(A)** and the GO function classifications **(B)** in *T. grandis*.

Supplementary Table 1 Changes of oil content with development of kernels in *T. grandis* ‘Xifei’ and *T. grandis* ‘Dielsii.’

Supplementary Table 2 Changes in content of FA composition with development of kernels in ‘Xifei’ and ‘Dielsii.’

Supplementary Table 3 Sequencing information of samples.

Supplementary Table 4 GO, KEGG, and Swissprot annotation of unigenes in *T. grandis*.

Supplementary Table 5 DEGs between *T. grandis* ‘Xifei’ and *T. grandis* ‘Dielsii.’

Supplementary Table 6 DEGs between developmental stages in *T. grandis* ‘Xifei.’

Supplementary Table 7 DEGs between developmental stages in *T. grandis* ‘Dielsii.’

Supplementary Table 8 The numbers of DEGs involved in pathways related to fatty acid biosynthesis and metabolism between *T. grandis* ‘Xifei’ and *T. grandis* ‘Dielsii.’

Supplementary Table 9 Primer sequences of DEGs used for RT-qPCR.

Supplementary Table 10 A total of 922 DEGs with *p*-value < 0.01 correlated to oil and fatty acids variation in *T. grandis* ‘Xifei.’

Supplementary Table 11 A total of 574 DEGs with *p*-value<0.01 correlated to oil and fatty acids variation in *T. grandis* ‘Dielsii.’

Supplementary Table 12 DETFs correlated to metabolites in *T. grandis* ‘Xifei’ and *T. grandis* ‘Dielsii’, respectively.

Supplementary Table 13 FPKM value of DEGs involved in pathway of FA syntheses used for heatmaps in *T. grandis* ‘Xifei’ and *T. grandis* ‘Dielsii’, respectively.
