# Supplementary figures and images for: Comparative de novo Transcriptome Analysis of Two Cultivars With Contrasting Content of Oil and Fatty Acids During Kernel Development in Torreya grandis
Source: Front Plant Sci. 2022 Jun 20;13:909759. doi: 10.3389/fpls.2022.909759 (PMC9251473; doi:10.3389/fpls.2022.909759)

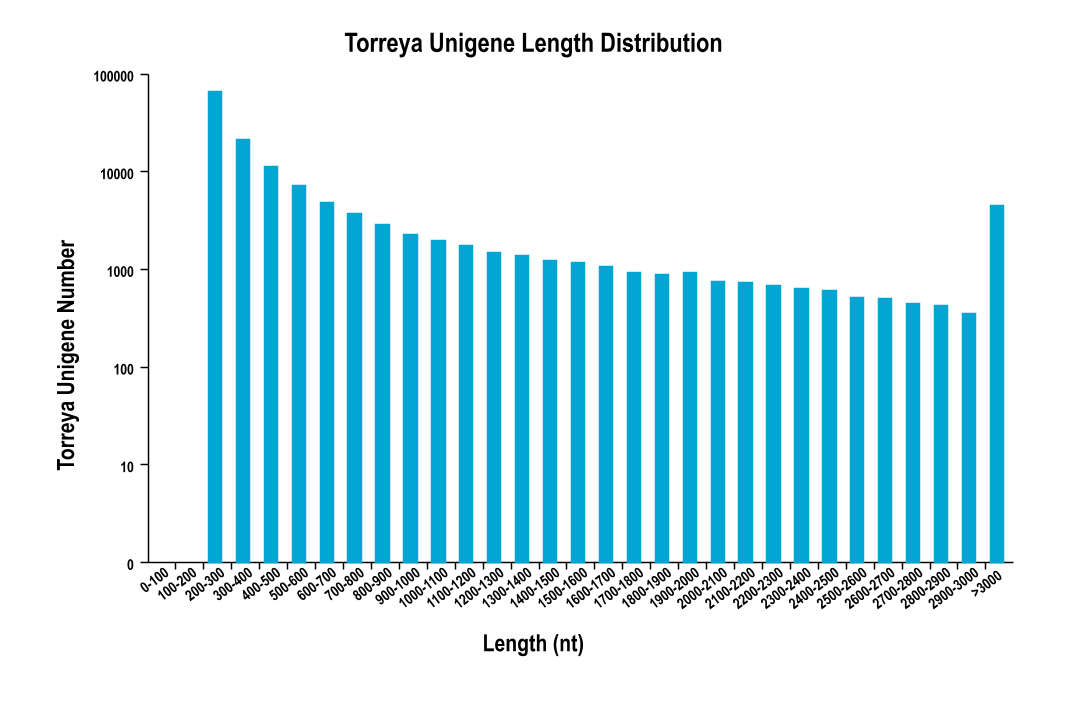

Supplement: Supplementary Figure 1 — Length distribution of unigenes with over 1 kb in length. [file Image_1.PNG]

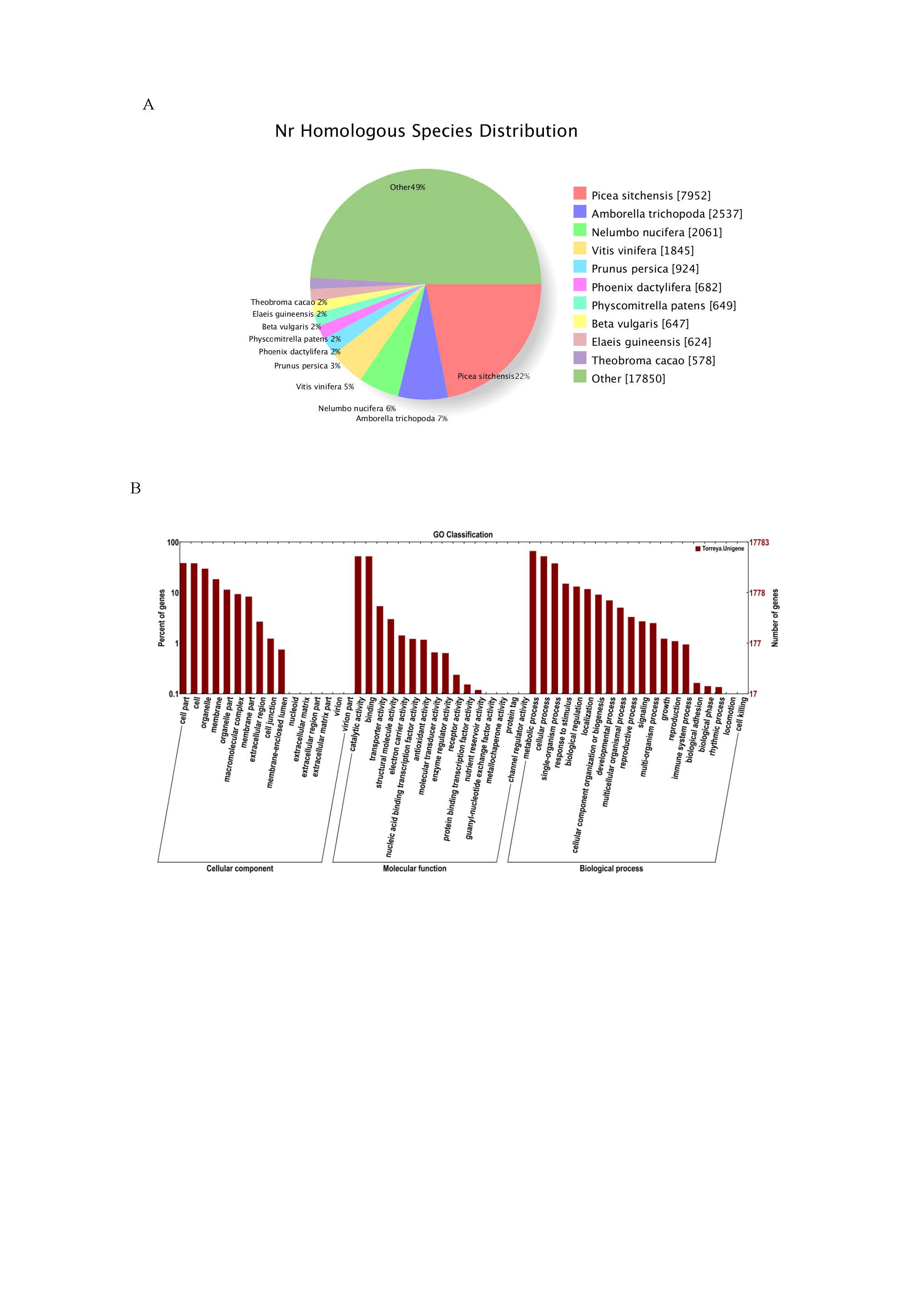

Supplement: Supplementary Figure 2 — The best-matched species of the unigenes (A) and the GO function classifications (B) in T. grandis. [file Image_2.JPEG]
